# The Cementation Mechanisms and Mechanical Properties of Different Soil–Rock Mixtures–Slurry Cements

**DOI:** 10.3390/ma18102186

**Published:** 2025-05-09

**Authors:** Jiayong Li, Zuliang Zhong, Hong Zou

**Affiliations:** 1 School of Civil Engineering, Chongqing University, Chongqing 400045, China; 20211601001z@stu.cqu.edu.cn (J.L.); 202316131450@stu.cqu.edu.cn (H.Z.); 2 National Joint Engineering Research Center of Geohazards Prevention in the Reservoir Areas, Chongqing University, Chongqing 400045, China

**Keywords:** soil–rock mixture, slurry type, rock content, mechanical properties, microscopic properties

## Abstract

This investigation focused on the cementation mechanisms and mechanical properties of soil–rock mixtures–slurry cement (SRM–SC) to ensure the safety of tunnels during operation. SRM–SC specimens were prepared with different types of slurry and rock contents based on an actual slurry injection ratio. The macroscopic level analysis involved measuring the specimens’ uniaxial compressive strength and shear strength, determining the strength parameters, and analyzing the damage forms. At the microscopic level, the surface morphology and composition of the specimens were examined using scanning electron microscope imaging. This allowed for a comparative analysis of the cementation ability and mechanism of the slurry under different control conditions, providing a basis for determining the mechanical properties of SRM–SC. The results indicated that the rock content significantly impacts the macromechanical properties of SRM–SC. The compressive strength and stiffness of SRM–SC initially increase and then decrease with the increasing rock content, with an inflection point observed between a 20% and 60% rock content. On the other hand, the shear strength and stiffness both increase with the increasing rock content. Additionally, the macroscopic mechanical properties of SRM–SC formed by different types of grout exhibit noticeable differences. These findings serve as a reference for regulating the mechanical properties of SRM–SC.

## 1. Introduction

A soil–rock mixture (SRM) is a highly heterogeneous geotechnical medium that formed during the Quaternary period. It consists of rock with a certain engineering scale and high strength, fine-grained soil, and void space [1]. The SRM is a geological body composed of rock as an aggregate and soil as a filling material [2]. The loose structure, large porosity, weak cementing ability, poor stability, and strong permeability of the backfill area of the soil–rock mixture often lead to deformation [3], uneven settlement, collapse, and water seepage in the cavern during tunneling through deeply buried backfill soil strata. These issues pose significant challenges to the project’s construction and can affect the regular operation of the tunnel [4,5]. Grouting reinforcement of the rock surrounding the tunnel is necessary to form a ring-shaped bearing arch in the soil layer around the hole, ensuring the stability of the hole and the tunnel face and improving the seepage-resistant performance of the rock surrounding the tunnel. Therefore, studying the cementation mechanism of soil–rock mixture grouting reinforcement, as well as the mechanical properties and the deformation damage mechanism after reinforcement, is of great theoretical significance and practical value for designing and constructing tunnel engineering projects.

Aiming at this particular geological body of soil–rock mixtures, many scholars have studied its physical–mechanical properties, infiltration characteristics, and deformation and damage mechanisms. The main research methods include in situ testing, laboratory testing [6], and numerical simulation [7,8]. In the study of the cementation mechanism and the physical–mechanical properties of slurry, Zhong et al. [9] explored the diffusion mechanism of modified cement slurry in pure soil and soil–rock mixtures using a self-developed modeling device. The experimental data were verified by deriving theoretical formulas for the diffusion radius. The soil–rock mixture–slurry cement (SRM–SC) is a discontinuous composite material consisting of soil, rock, slurry, and voids.

Avsar [10] quantitatively characterized the geometrical and structural properties of mélanges using direct shear tests, digital image processing, and fractal analyses. Wang et al. [11] investigated the non-Darcy seepage characteristics using Forchheimer’s equation and obtained the permeability coefficients of SRMs at different block percentages. The indoor flow tests showed that the permeability coefficient of SRM with a clay matrix increased with the increase of the hydraulic gradient [12,13]. In the study of the dynamic characteristics of soil–rock mixtures, Zhong et al. [14] employed dynamic triaxial testing to examine the dynamic behavior of soil–rock mixture backfills under the influence of cyclic loads generated by subway train traffic. The study’s findings indicate that the softening index exhibits a more rapid decay and a greater amplitude of deformation with an increase in the number of loading cycles, particularly when the initial degree of consolidation and the effective confining stress are lower. Huang et al. [15] explored the effects of the stone content, water content, consolidation stress ratio, and vibration frequency on the morphological characteristics of the hysteresis loops in soil–rock mixtures. Their research revealed that an increase in the stone content, consolidation stress ratio, and loading frequency corresponded with a reduction in the distance between the centers of the hysteresis loops, indicative of a decrease in plastic deformation. Wang et al. [16] utilized the Global Digital Systems (GDSs) dynamic triaxial testing system to investigate the response of typical coarse-grained soil fillers to step cyclic loading, analyzing the influence of different confining pressures, consolidation ratios, and the number of loading cycles on the hysteresis loop characteristics. The results demonstrated that confining pressure plays a significant role in reducing the energy dissipation of soil samples under cyclic loading. Existing rock strength prediction equations were mainly applied to jointed rock bodies based on the strength damage criterion proposed by Tian et al. (2018) [17]. Therefore, Zhang et al. [18] studied the nonlinear relationship between the shear strength of SRMs and the proportion of rock blocks (RBPs) and proposed an empirical formula for predicting the shear strength of firm rock–weak soil mixed materials. Wang et al. [19] found that the peak shear force increases with the size of the rock, and when the rock size is larger, the interlocking and locking phenomenon of the specimens becomes more pronounced. Qian et al. [20] established a 2D numerical computational model of soil–rock mixtures using a Particle Flow Code (PFC) and a Fast Lagrangian Analysis of Continua (FLAC) to characterize the mechanical features of soil–rock mixtures at the fine-scale level. A soil–rock mixture containing irregularly shaped rock clasts was established based on a self-developed 3D discrete element technique [21,22]. Their results showed that the strength of SRM–SC depends on the block skeleton effect and the cementation effect. Park and Kim [23] prepared ten mixed-ratio slurries using different combinations of materials, such as ordinary Portland cement, ultrafine cement, biological grouting material, and sodium silicate aqueous solution. The test results showed that a 30% proportion of biological grouting material can be used instead of cement to save costs during grouting [24]. The effects of naphthalene water-reducing agents and polycarboxylic acid water-reducing agents on the setting time, mobility, and compressive strength of sulfur–aluminate cements were investigated [25]. Ma and Liu [26], through a scanning electron microscope analysis of the microtopography of rock joint surfaces and research on the interfacial transition zone, derived the micromechanism of bond shear failure between cement paste and a rock interface, without considering the effect of macroscopic roughness. Tian et al. [27] carried out experimental and numerical studies, and a mechanical model of cement concrete–rock cemented surfaces describing the bonds’ elastic behavior before reaching peak shear stress and the post-peak behavior due to bond failure and increased friction was proposed. The geomechanical parameters of composite media cement were evaluated using unconventional in situ shear and sieve tests and wave velocity testing methods [28,29]. Related studies in the field of concrete [30,31] had shown that ordinary Portland cement slurries tended to be characterized by high porosity and the low strength of the cement slurry formed by them due to the large cement particle size, which resulted in insufficient hydration reactions.

While existing studies have predominantly focused on the influence of macroscale variables, such as the rock content and gradation, on the mechanical properties of soil–rock mixtures (SRMs), there remains a critical knowledge gap in systematically elucidating the microscopic cementation mechanisms associated with different slurry types (e.g., ordinary Portland cement, low-alkalinity sulfoaluminate cement). Conventional methodologies relying on macroscopic mechanical tests (e.g., uniaxial compression, direct shear tests) merely establish input–output correlations, failing to unravel microscale governing mechanisms, such as the spatial distribution of hydration products or the evolution of microcracks within interfacial transition zones (ITZs). The inherent heterogeneity and anisotropy of SRMs result in mechanical responses that significantly deviate from continuum medium assumptions. Current macroscale constitutive models inadequately quantify the dynamic interplay between slurry–soil–rock interfacial debonding and rock skeleton interlocking effects, thereby limiting the predictive accuracy in engineering applications. To address these limitations, this study employs scanning electron microscopy (SEM) and energy-dispersive spectroscopy (EDS) to characterize the formation patterns of hydration products and microporous filling characteristics at soil–rock interfaces for three slurry types: ordinary Portland cement (OPC), low-alkalinity sulfoaluminate cement (LASC), and OPC–water–glass composites. These microscale observations are systematically correlated with macroscopic mechanical performance metrics to establish causal linkages between cementation quality and bulk mechanical behavior.

In summary, the current experimental research on the mechanical properties of cemented soil–rock mixtures is insufficient. Therefore, this paper aims to investigate the effects of the rock content and slurry type on the mechanical properties of cemented soil–rock mixtures. We used soil samples from the interval tunnel from Wangjiacheng Station to Shengjiabao Station of Chongqing Railway Transportation Line 4, Phase II Civil Construction Standard 5, to prepare SRM–SC specimens with different rock contents and slurry types by adding water and cement. We conducted uniaxial compression tests, direct shear tests, and microcosmic characterization tests. The analysis focused on the influence of the rock content and slurry type on the mechanical properties of the cemented soil–rock mixture. Furthermore, the influence of the microscopic mechanisms of the slurry type was deeply analyzed, including the microcrack evolution, cement filling capacity, and the content of calcium silicate hydrate gel. The findings of this study can provide valuable insights for engineering practice.

## 2. Experiments on the Macromechanical Properties and Microscopic Characterization of SRM–SC

### 2.1. Macromechanical Property Experiment on SRM–SC

#### 2.1.1. Test Materials

The test materials consisted primarily of ordinary Portland cement, low-pH-value sulfoaluminate cement, water–glass, water, and a soil–rock mixture.

(1) Ordinary Portland cement

Ordinary Portland cement produced by Jidong Cement Chongqing Hechuan Limited Liability Company (Chongqing, China), was used, with a code strength grade of P.O 42.5, and the fundamental performance indexes are shown in Table 1. The fundamental performance indicators of ordinary Portland cement are provided by cement producers.

(2) Low-alkalinity sulfoaluminate cement

Emeishan Qianghua Special Cement Co., Ltd. (Emeishan City, Sichuan, China), produced low-alkalinity sulfoaluminate cement with the code strength grade of L–SAC42.5, and the fundamental performance indexes are shown in Table 2. The fundamental performance indicators of low-alkalinity sulfoaluminate cement were provided by cement producers.

(3) Water–glass

The water–glass produced by the Chongqing Bishan Yongsheng Water–Glass Co., Ltd. (Chongqing, China), was used, and the fundamental performance indexes are shown in Table 3. The fundamental performance indicators of water–glass were provided by water–glass producers.

(4) Soil–rock mixture

The soil–rock mixture was taken from the soil–rock mixture backfill area under section YK47 + 780.110~YK48 + 011.110 of the tunnel from Wangjiacheng Station to Shengjibao Station of Chongqing Railway Transportation Line 4, Phase II Civil Construction 5 Standard, with argillaceous sandstone as the main rock and silty clay as the fine-grained soil. The fundamental physical parameters of the soil–rock mixture are shown in Table 4. The parameter values presented in Table 4 were obtained from the geological investigation report. Through laboratory geotechnical tests, we can obtain the basic physical parameters of silty clay. The specific process is as follows:

Moisture content determination: The moisture content can be calculated by measuring the mass change of silty clay before and after drying. First, place the soil sample in an oven and dry it at 105 °C to a constant weight to obtain the dry soil mass. Then, divide the mass difference before and after drying by the dry soil mass to obtain the moisture content.

Density determination: The density of silty clay is determined using the cutting ring method. First, place the soil sample into a cutting ring of known mass, level the surface, and weigh the total mass of the cutting ring and soil sample. Then, subtract the mass of the cutting ring from the total mass to obtain the mass of the soil sample. Finally, divide the mass of the soil sample by the volume of the cutting ring to obtain the density.

Specific gravity determination: the specific gravity of silty clay is determined using the pycnometer method.

Volume determination: The volume of silty clay is measured using a measuring cylinder. First, add a certain amount of water to the measuring cylinder and record the initial water level. Then, slowly add the soil sample of known mass to the measuring cylinder and record the final water level. By calculating the change in water level, the volume of the soil sample can be obtained.

Based on the above data, we can calculate the void ratio of silty clay. The void ratio is the ratio of the volume of voids to the volume of solid particles in the soil.

The soil and rock threshold was the boundary of the particle size of “soil” and “rock” in the soil–rock mixture, which was an essential physical property index reflecting the scale independence of the soil–rock mixture. According to the Standard for Geotechnical Test Methods (GB/T50123–2019) [32], 5 mm was selected as the soil and rock threshold, and the maximum particle size of the rock was not more than 60 mm. P5 indicates the size of the rock content in the backfill of the soil–rock mixture [33].

**Figure 1:**
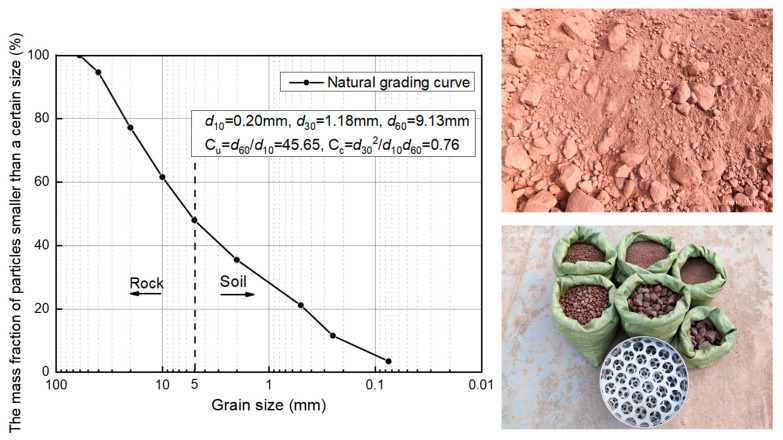
Gradation curve of soil–rock mixture backfill samples taken from the site.

The on-site soil–rock mixture backfill has a mass and volume exceeding the test conditions of the super-granular rock; any particle size of greater than 60 mm of rock was removed, and a sieve test was performed to obtain the field soil–rock mixture gradation curves, as shown in Figure 1. The subsequent test, the soil–rock mixture backfill, was also used in Figure 1 of the gradation for the preparation.

#### 2.1.2. Preparation Program for SRM–SC

The SRM–SC taken out on site was affected by disturbance; the soil wrapped by the slurry–rock skeleton was dislodged, and it was impossible to cut standard-size specimens by the waterjet cutting method. In this study, the amount of grouting material needed per unit grouting volume was calculated based on the actual engineering slurry injection ratio of 150–200 kg of cement per unit depth of grouting and the hole arrangement of 1.2 m plum blossom piles, and the homemade SRM–SC was approximated instead of measuring the actual grouted reinforced cement.

(1) Testing equipment

The leading equipment used to make the specimens of SRM–SC was an electronic balance, shaking table, mixing drum, vibrator, trowel, standard sieve, molds, lubricant, and curing chamber (provided at the construction site).

(2) Testing programs

The tests considered the effects of the slurry type, rock content, and axial pressure on the mechanical properties of SRM–SC, and the test programs shown in Table 5 and Table 6 were formulated. Specimens 1–9 were designed to study the effects of using different types of slurry injection reinforcement and different axial pressures on the shear strength of SRM–SC. The types of slurry were an ordinary Portland cement single-liquid slurry; a low-alkalinity sulfoaluminate cement single-liquid slurry; and an ordinary Portland cement–water–glass double-liquid slurry (the volume ratio of cement to water–glass was 1:1), and the slurry water–cement ratios were all 1:1, starting now referred to as slurry A, slurry B, and slurry C, respectively. Specimens 10–11 were used to study the effect of the rock content on the shear strength of SRM–SC, and the rock content in the field ranged from 20% to 50%, so the three levels of the rock content were taken as 20%, 40%, and 60%, respectively. The axial pressure levels of the shear test were taken as 0.6 MPa, 0.8 MPa, and 1.0 MPa, respectively. Specimens a–i were taken to study the effects of different types of slurry reinforcement and different rock contents on the compressive strength of the SRM–SC.

(3) Specimen preparation of SRM–SC

**Figure 2:**
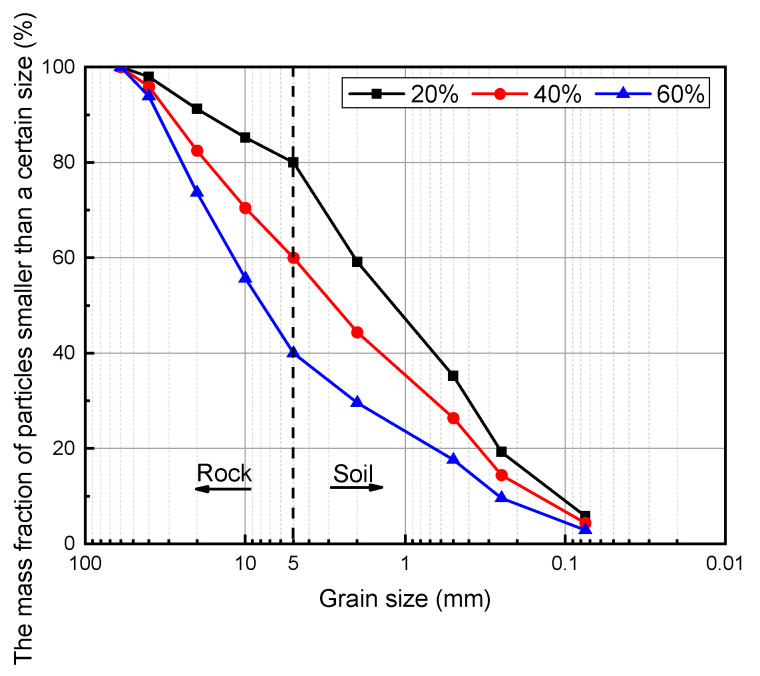
Design gradation curves of soil–rock mixture backfills with different rock contents.

According to the grading and the rock content in Figure 2, the silty clay and rock were weighed and introduced into the mold at one time, and then the necessary amount of water was added uniformly and thoroughly mixed according to the natural moisture content of the soil–rock mixture. The grading curves of the soil–rock mixture backfill specimens with different rock contents are shown in Figure 2. The uniformly mixed slurry was injected into the center of a standard mold filled with the soil–rock mixture backfill, which was then placed on a vibrating table for vibratory consolidation. In the course of specimen fabrication, the grouting procedure was completed within a duration of 5 min. Subsequently, the vibration consolidation was executed for a period of 1 min. Following a 24 h curing period in the standard curing chamber, the samples were demolded and subjected to a further 28 days of curing. The standard curing chamber was maintained at a temperature of 20 °C and a relative humidity of 96%. Each test group contained three parallel specimens and a total of 33 direct shear specimens with a length × width × height of 150 mm × 150 mm × 150 mm, and 27 uniaxial compression specimens with a diameter × height of Φ50 mm × 100 mm were fabricated in this test.

#### 2.1.3. Test Procedure

**Figure 3:**
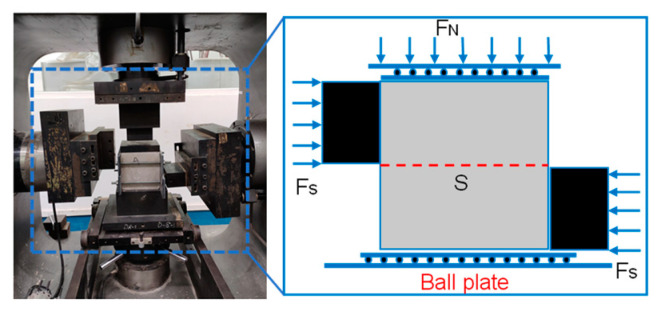
The state of the completed sample installation: direct shear test.

Conventional uniaxial compression tests and direct shear tests were carried out on SRM–SC specimens of different types of slurries (slurries A, B, and C) with different rock contents (20%, 40%, and 60%), as shown in Figure 3. The tests mainly consisted of the following steps:

1. The installation and commissioning of the shear head of the testing machine. Different-sized vertical shear heads were used for uniaxial compression tests and direct shear tests; the heights and cross-sectional dimensions of the horizontal shear heads on both the left and right sides were adjusted to fit the size of the self-made shear box. The self-made shear box consisted of an upper box and a lower box, each made of two steel plates with the dimensions of length × width × thickness = 25 cm (35 cm) × 7 cm × 1 cm, which were connected using lead screws and bolts.

2. The installation of test specimens. Prior to testing, the surface of the specimen must be polished with sandpaper to achieve the standard flatness. Subsequently, an appropriate amount of Vaseline was applied to the areas where the specimen comes into contact with the apparatus or shear box. Additionally, to prevent damage from self-contact between various parts of the instrument, a rigid block with a base area approximately equal to the upper surface area of the specimen was placed on top of the SRM–SC to uniformly distribute the normal load. This ensures that the specimen is centered with the vertical shear head to prevent any bias and measurement errors during shearing. The bottom of the specimen was equipped with a ball bearing plate, which ensured that the shear force measured by the instrument primarily originated from the shear failure plane. The completed specimen installation is shown in Figure 3. The direct shear equation is $\tau =\frac{F_{s}}{S}$, where $F_{s}$ is the shear force, S is the shear area, and τ is the shear stress.

3. Axial loading. Initially, the axial loading was pre–applied in displacement control mode until the vertical shear head was just about to make contact with the upper part of the specimen, at which point, the axial force began to increase and the loading was stopped. The force and displacement values for the axial direction were then reset to zero. Subsequently, the formal axial compression loading was applied using a force control method for both the uniaxial compression tests and the direct shear tests, with a loading rate of 0.01 kN/s. The loading was maintained at the specified axial stress level after reaching it.

4. Shear loading. Initially, a preliminary horizontal loading was conducted in a displacement-controlled mode, ceasing when the right horizontal shear head was on the verge of contacting the shear box, as indicated by the onset of an increase in shear force values. Thereafter, the definitive shear loading was initiated, employing a displacement-controlled approach with a loading rate of 0.3 mm/min.

5. The acquisition of data. For each experimental condition, three specimens were tested, and the final result was the average of the three test outcomes. Axial and shear displacements, along with the corresponding forces, were continuously monitored and automatically recorded throughout the testing process using the WDAJ-600 rock shear rheometer (ChangChun Keyi Test Instrument Co., Ltd, Jilin, China).

### 2.2. Microscopic Characterization Experiment of SRM–SC

The macroscopic mechanical properties of SRM–SC are inextricably linked to their internal microstructures. To compare and analyze the cementation ability and cementation mechanism of different slurry–soil–rock mixtures, scanning electron microscopy (SEM) equipped with energy dispersive X–ray spectroscopy (EDX) was used to analyze the surface morphology and composition of typical debris specimens after macromechanical property tests.

**Figure 4:**
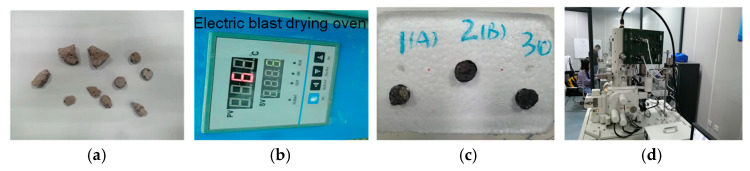
The primary test process of scanning electron microscopy: (**a**) Sample selection. (**b**) Clean and dry. (**c**) Surface gilding (A, B, and C represent slurry A, slurry B, and slurry C). (**d**) Vacuum scanning.

The main process of the test is shown in Figure 4. The test consists of the following main steps:

1. The collection of the SRM–SC specimens left following the mechanical tests. From these specimens, a fragment specimen with a flat bottom and a size adapted to the size of the sample table (the size of the sample table was Φ12.7 × 9 mm) was selected as the sample to be tested; the specimen contained small rock particles (the particle size was approximately 5 mm), presenting the interface between the slurry and the rock. One test sample was made for each of the three grouting materials.
2. Cleaning and drying treatment. To ensure the actual uncontaminated state of the specimen surface, the slices were soaked in anhydrous alcohol for 1 d to terminate hydration and then dried at 65 °C to a constant weight.
3. Surface gilding. For samples with poor electrical conductivity, such as SRM–SC, it was necessary to spray a conductive layer before observation to form a conductive film on the surface of the material to avoid the accumulation of charge on the surface of the sample, to improve the quality of the image and to prevent thermal damage to the sample. The samples were pasted onto the scanning electron microscope sample stage with professional conductive adhesive, and the surface was plated with a spraying rate of 30 s × 4 times.
4. Upon attaining a vacuum level of lower than $10^{-4}$ Torr, we proceeded with the electron microscope scanning analysis.

## 3. The Analysis of the Results of the SRM–SC Microscopic Property Detection Test

### 3.1. The Analysis of the Electron Microscope Scanning Test Results of SRM–SC

**Figure 5:**
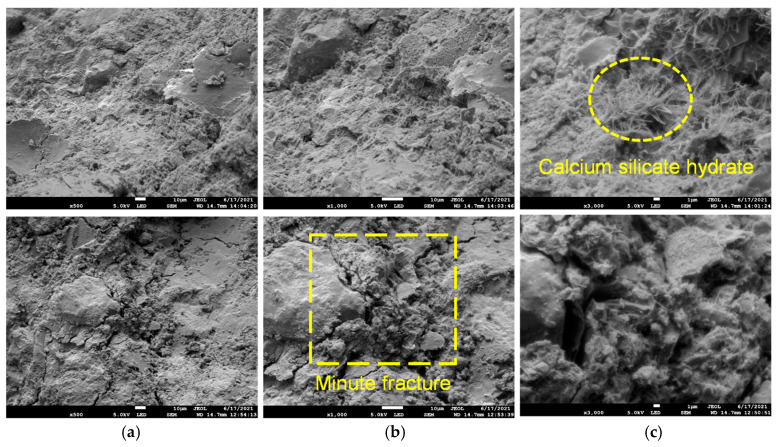
Microstructure of ordinary Portland cement slurry cementation (WD = 14.7 mm, EHT = 5.0 KV): (**a**) amplification factor (500); (**b**) amplification factor (1000); (**c**) amplification factor (3000).

Shear damage fragments of SRM–SC formed by different types of slurry reinforcement were selected to observe the degree of the densification of the cemented surface, the size, shape, and distribution of pores, the structural morphology of the hydration products, and the bonding condition at the interface of the rock and rock materials. Figure 5 shows the microstructure of the cementation of ordinary Portland cement slurry, from which it can be seen that

1. There were defects such as tiny cracks on the cemented surface, interconnected pores between the calcium silicate hydrate gel ($CaO\cdot nSiO_{2}\cdot mH_{2}O$) particles, voids between the hydration products, capillary pores that were not filled by the hydration products, and voids between the soil particles, and the defects were obviously distributed in a wide range and large in number;
2. The calcium silicate hydrate showed a fiber-like and flocculent, sparse distribution in the vicinity of voids and minute fractures;
3. The distribution of calcium silicate hydrate on the surface of different scanning areas varied in density, and the distribution of calcium silicate hydrate in the same scanning area was not uniform;
4. The voids between the interface of the rock particles and other particles were filled with calcium silicate hydrate, but the number of hydration products produced was not enough to fill the entire void, and the parts without cementation could still be clearly seen. The particles were more loosely connected, without forming a solid cementation.

**Figure 6:**
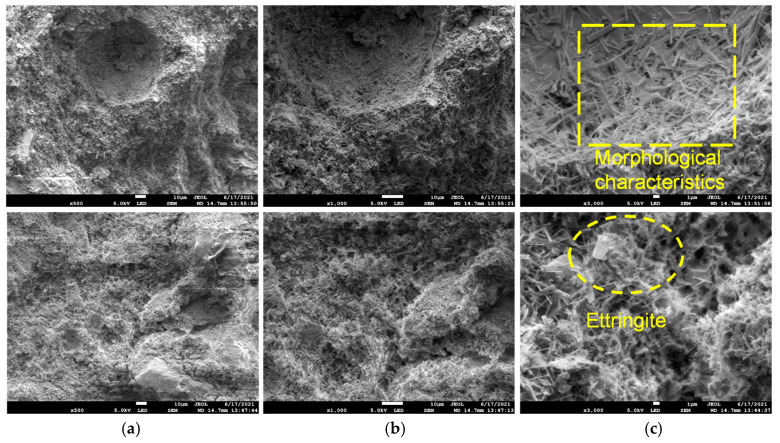
Microstructure of cementation of low-alkalinity sulfoaluminate cement slurry (WD = 14.7 mm, EHT = 5.0 KV): (**a**) amplification factor (500); (**b**) amplification factor (1000); (**c**) amplification factor (3000).

Figure 6 shows the microstructure of the low-alkalinity sulfoaluminate cement slurry cementation, as seen in the figure:

1. Compared with the cement surface of the ordinary Portland cement slurry, the cement surface of the low-alkalinity sulfoaluminate cement slurry was basically free of defects, such as minute fractures, and was mainly dominated by interconnected pores between ettringite ($3CaO\cdot Al_{2}O_{3}\cdot 3CaSO_{4}\cdot 32H_{2}O$) particles.
2. The main product of hydration was ettringite, with minor amounts of calcium silicate hydrate and hydrated aluminum oxide. Ettringite was densely distributed in columnar and cluster shapes, interweaving and cementing with each other to form a net-like structure that filled and covered the entire scanning area. Calcium silicate hydrate was dispersed within it in fibrous and flocculent forms.
3. The distribution of hydration products on the surface of different scanning areas was approximately the same density, and the distribution of hydration products in the same scanning area was uniform.
4. Besides the hydration products, such as ettringite and calcium silicate hydrate, on the cemented surface, no soil particles or rock particles could be seen.

**Figure 7:**
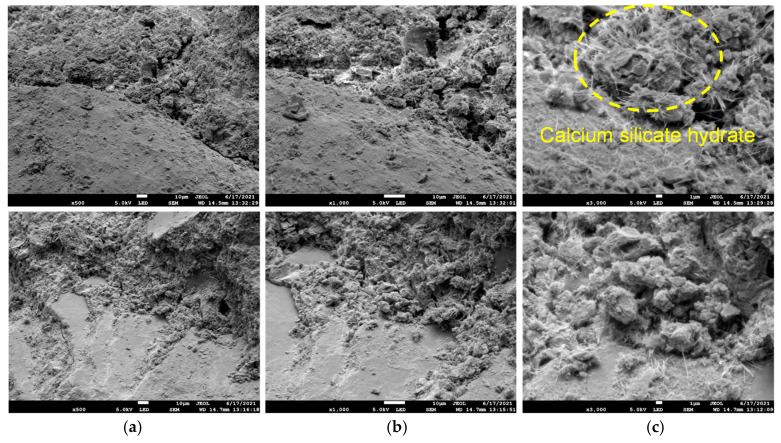
Microstructure of ordinary Portland cement–water–glass slurry cementation (WD = 14.7 mm, EHT = 5.0 KV): (**a**) amplification factor (500); (**b**) amplification factor (1000); (**c**) amplification factor (3000).

Figure 7 shows the microstructure of ordinary Portland cement–water–glass slurry cementation, as seen in the figure:

1. The defects on the cemented surface of ordinary Portland cement–water–glass slurry were fewer and mainly dominated by the voids between the hydration products ($CaO\cdot nSiO_{2}\cdot mH_{2}O$).
2. The calcium silicate hydrate showed a fibrous and flocculent distribution near the interface between the voids and the particles of the rock mass, and the generation of the calcium silicate hydrate was significantly increased in number and densely distributed compared to that on the cemented surface of the ordinary Portland cement.
3. The distribution of hydration products on the surface of different scanning areas had approximately the same density, and the distribution of hydration products in the same scanning area was uniform.
4. The calcium silicate hydrate filled the voids and surfaces covered between the interfaces of the rock particles.

### 3.2. Analysis of the Reinforcement Mechanism of SRM–SC

#### 3.2.1. The Principle of the Hydration Reaction of Ordinary Portland Cement

The mineral composition of ordinary Portland cement clinker is dominated by tricalcium silicate ($C_{3}S$), dicalcium silicate ($C_{2}S$), tricalcium aluminate ($C_{3}A$), and tetracalcium ferroaluminate ($C_{4}AF$), and their respective hydration chemical formulas are shown in Equations (1)–(4). Among them, the calcium silicate hydrate ($CaO\cdot nSiO_{2}\cdot mH_{2}O$) generated by the hydration reaction of $C_{3}S$ and $C_{2}S$ plays the role of gelling and finally forms the strength. The hydration reaction of $C_{3}S$ is relatively fast, which is related to the formation of early strength, while the hydration reaction of $C_{2}S$ is relatively slow, which is crucial for forming late strength.

(1) $$3CaO\cdot SiO_{2}+nH_{2}O\to xCaO\cdot SiO_{2}\cdot yH_{2}O+(3-x)Ca(OH{)}_{2}$$

(2) $$2CaO\cdot SiO_{2}+nH_{2}O\to xCaO\cdot SiO_{2}\cdot yH_{2}O+(2-x)Ca(OH{)}_{2}$$

(3) $$3CaO\cdot Al_{2}O_{3}+26H_{2}O+3\left(Ca{SO}_{4}\cdot 2H_{2}O\right)\to 3CaO\cdot Al_{2}O_{3}\cdot 3Ca{SO}_{4}\cdot 32H_{2}O$$

(4) $$4CaO\cdot Al_{2}O_{3}\cdot Fe_{2}O_{3}+27H_{2}O+3\left(Ca{SO}_{4}\cdot 2H_{2}O\right)+Ca(OH{)}_{2}\;\to 3CaO\cdot Al_{2}O_{3}\cdot 3Ca{SO}_{4}\cdot 32H_{2}O+Ca_{2}Fe_{2}O_{5}\cdot 2H_{2}O$$

**Figure 8:**
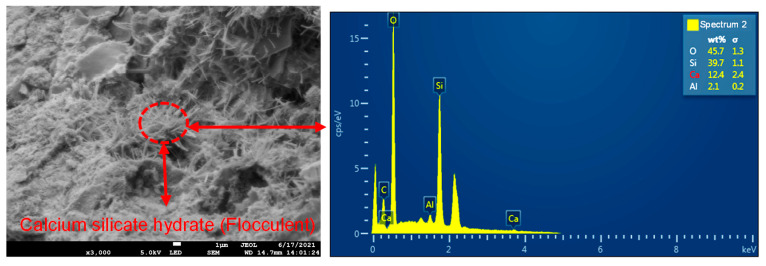
EDS analysis of ordinary Portland cement slurry cementation (WD = 14.7 mm, EHT = 5.0 KV, amplification factor 3000).

The EDS energy spectrum analysis of the ordinary Portland cement slurry cementing surface corresponding to the scanning position is given in Figure 8, which shows that the main constituent elements were Ca, O, Si, and Al. The elemental weights of spectrum 2 were Ca (12.4%), O (45.7%), Si (39.7%), and Al (2.1%). This suggests that this fibrous and flocculent hydration product was calcium silicate hydrate.

#### 3.2.2. The Principle of the Hydration Reaction of Low-Alkalinity Sulfoaluminate Cement

The mineral composition of the low-alkalinity sulfoaluminate cement clinker is dominated by anhydrous calcium sulfoaluminate ($C_{4}A_{3}S$) and dicalcium silicate ($C_{2}S$). $C_{4}A_{3}S$ reacts with gypsum to produce ettringite ($3CaO\cdot Al_{2}O_{3}\cdot 3CaSO_{4}\cdot 32H_{2}O$) and hydrated alumina ($Al_{2}O_{3}\cdot 3H_{2}O$); $C_{2}S$ hydrates to produce calcium silicate hydrate; a $Ca{\left(OH\right)}_{2}$ and hydrated alumina and gypsum reaction continues to generate ettringite; the hydration reaction are shown in Equations (5)–(7). Ettringite was the main hydration product that played the role of dense filling, and the calcium silicate hydrate and hydrated alumina played the role of gelling.

(5) $$3CaO\cdot 3Al_{2}O_{3}\cdot CaSO_{4}+2(CaSO_{4}\cdot 2H_{2}O)+34H_{2}O\;\to 3CaO\cdot Al_{2}O_{3}\cdot 3CaSO_{4}\cdot 32H_{2}O+2(Al_{2}O_{3}\cdot 3H_{2}O)$$

(6) $$2CaO\cdot SiO_{2}+2H_{2}O\to CaO\cdot SiO_{2}\cdot H_{2}O+Ca(OH{)}_{2}$$

(7) $$Al_{2}O_{3}\cdot 3H_{2}O+3Ca{\left(OH\right)}_{2}+3(CaSO_{4}\cdot 2H_{2}O)+20H_{2}O\;\;\;\;\;\;\;\;\;\;\;\;\;\;\;\;\;\;\to 3CaO\cdot Al_{2}O_{3}\cdot 3CaSO_{4}\cdot 32H_{2}O$$

**Figure 9:**
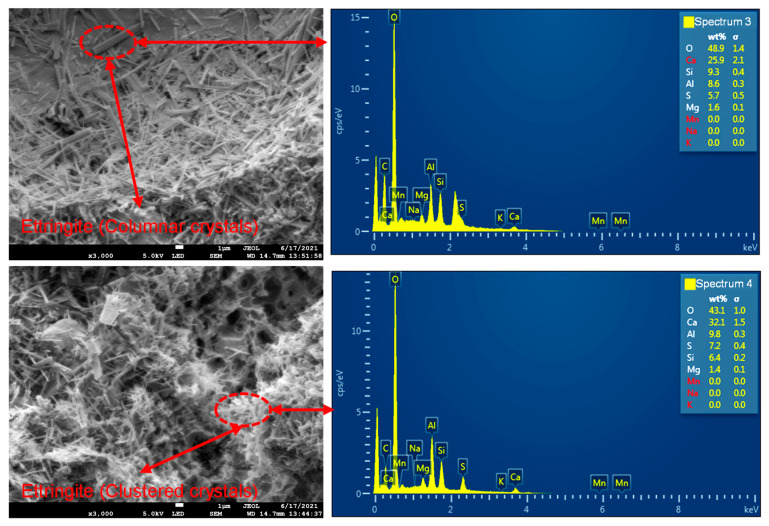
EDS analysis of low-alkalinity sulfoaluminate cement slurry cementation (WD = 14.7 mm, EHT = 5.0 KV, amplification factor 3000).

The EDS energy spectrum analysis of the low-alkalinity sulfoaluminate cement slurry cementing surface corresponding to the scanning position is given in Figure 9. Through the EDS energy spectrum analysis, it can be seen that the primary constituent elements were Ca, O, Si, S, and Al. Among them, the quantitative amounts of the elements in spectrum 3 were Ca (25.9%), O (48.9%), Si (9.3%), Al (8.6%), and S (5.7%); the quantitative amounts of elements in spectrum 4 were Ca (32.1%), O (43.1%), Si (6.4%), Al (9.8%), and S (7.2%); and other elements that may be present were Mg, Mn, Na, and K, which were presumed to be from the elements in the mineralogical composition of the soil–rock mixture. From the chemical composition, the columnar and clustered crystals on the cementing surface of the low-alkalinity sulfoaluminate cement slurry were known to be ettringite.

#### 3.2.3. The Principle of the Hydration Reaction of Ordinary Portland Cement–Water–Glass

The hydration reaction of the ordinary Portland cement–water–glass slurry is based on the hydration reaction of ordinary Portland cement plus the reaction of $Ca{\left(OH\right)}_{2}$ with the water–glass aqueous solution, as shown in Equation (8). From Equation (8), it can be seen that the $Ca{\left(OH\right)}_{2}$ generated by the hydration reaction of $C_{3}S$ and $C_{2}S$ in ordinary Portland cement will react with a water–glass aqueous solution to continue to generate calcium silicate hydrate with a gelling effect, and the generated products’ consumption will accelerate the hydration reaction rate of the ordinary Portland cement. Therefore, adding a water–glass aqueous solution not only accelerates the speed of the hydration reaction and causes the reaction to achieve high strength earlier but also generates additional calcium silicate hydrate so that the cementing ability can be enhanced.

(8) $$Ca{\left(OH\right)}_{2}+{Na}_{2}O\cdot nSiO_{2}+mH_{2}O\to CaO\cdot nSiO_{2}\cdot mH_{2}O+2NaOH$$

**Figure 10:**
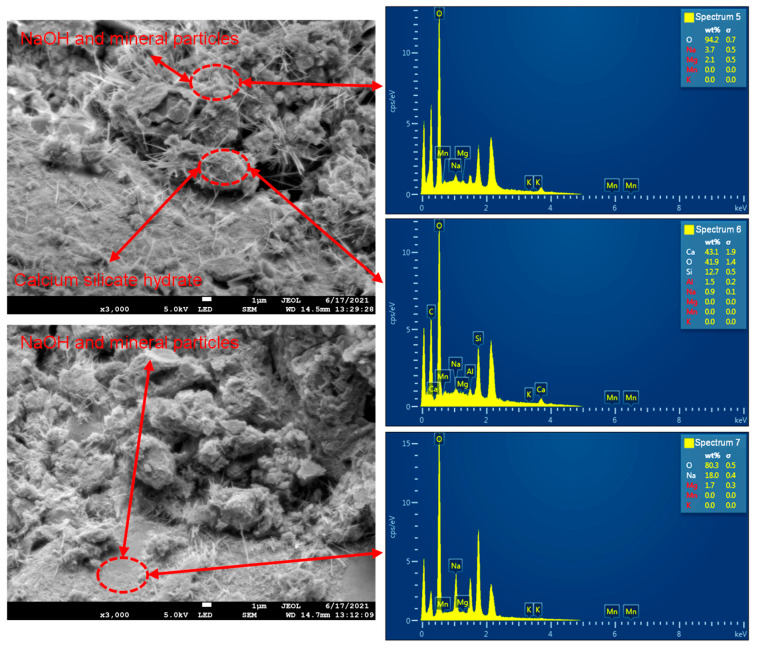
EDS analysis of ordinary Portland cement–water–glass slurry cementation (WD = 14.7 mm, EHT = 5.0 KV, amplification factor 3000).

The EDS energy spectrum analysis of the ordinary Portland cement–water–glass slurry cementing surface corresponding to the scanning position is given in Figure 10. Through the EDS energy spectrum analysis, it can be seen that the primary constituent elements were Ca, O, Si, Al, and Na. Among them, the quantitative amount of spectrum 5 was Na (3.7%) and O (94.2%), respectively; the quantitative amount of spectrum 6 was Ca (43.1%), O (41.9%), Si (12.7%), and Al (1.5%); the quantitative amount of spectrum 7 was Na (18.0%) and O (80.3%); and the other possible elements were Mg, Mn, and K, which were presumed to come from the elements in the mineralogical composition of the soil–rock mixture. From the chemical composition, it can be seen that the fibrous and flocculent material in Figure 10 was the main hydration product, calcium silicate hydrate, and contained the alkaline material NaOH, which was generated by reacting with the aqueous solution of water–glass and the possible presence of mineral particles of SRM–SC.

However, evaluating the effect of grouting reinforcement only from the microscopic characterization of SRM–SC is not comprehensive. In order to more comprehensively analyze and deal with the engineering problems of the grouting reinforcement of soil–rock mixture backfill soil stratum, this paper will discuss the intrinsic factors of slurry types and rock contents affecting the macromechanical property of SRM–SC at the macroscopic level in Section 5.

## 4. The Analysis of the Results of the Macroscopic Mechanical Property Test of SRM–SC

### 4.1. The Analysis of the Uniaxial Compression Test Results

The arithmetic mean values of uniaxial compressive strength and peak strain for SRM–SC are given in Table 7. As evidenced by Table 7, both the slurry types and the rock contents exert significant influences on the compressive strength and peak strain of SRM–SC. In general, the compressive strengths of slurry A, B, and C exhibit an initial increase followed by a subsequent decline as the rock content rises. Notably, slurry C demonstrates the highest compressive strength among the three slurries, reaching a maximum value of 4.36 MPa at the 40% rock content, which underscores its superior cementation efficacy under this specific rock proportion. Regarding the peak strain, slurries B and C display a consistent decreasing trend with the increasing rock content, whereas slurry A exhibits a unique pattern characterized by an initial reduction followed by an augmentation in peak strain. This divergence highlights the pronounced variability in deformation capacity across different slurry–rock content combinations.

### 4.2. The Analysis of the Direct Shear Test Results

The arithmetic mean values of shear strengths and corresponding peak shear displacements for the SRM–SC are given in Table 8.

**Figure 11:**
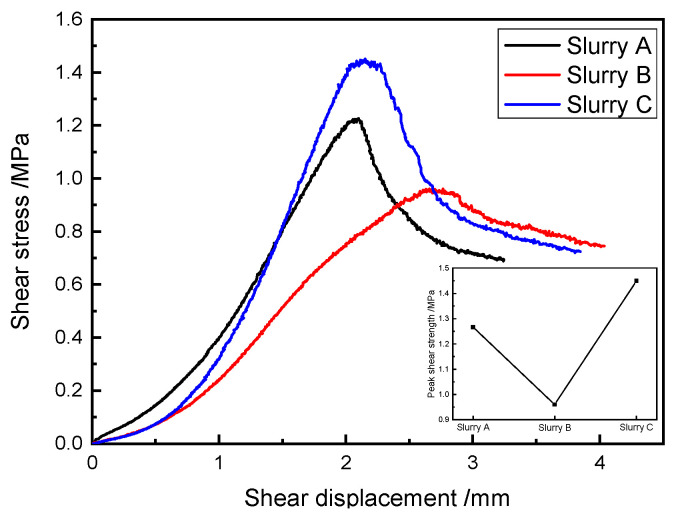
The shear stress–displacement curves for the SRM–SC (rock content 40%, axial pressure $\delta_{N}=0.6\;MPa$).

Figure 11 shows the shear stress–shear displacement curves of the SRM–SC specimens under the current test conditions. The shear process was divided into four stages: compaction, linear, nonlinear, and post-peak softening.

In the compaction stage, the SRM–SC specimen was placed under the action of axial pressure and lateral shear force, and the original small voids and fractures were compacted, and the internal yielding or damage did not occur locally; the shear stress–shear displacement curve presented a downward concave shape, and the slope of the curve increased; that is, the shear stiffness gradually increased. In the linear stage, the specimen underwent elastic deformation, and the stage of the shear stress increased steadily, resulting in a large amount of linear strain accumulation, but did not reach the ultimate shear strength. In the nonlinear stage, the shear stress–shear displacement curve presented an upward convex shape, and the slope of the curve decreased; that is, the shear stiffness decreased, and the shear stress increased slowly and finally reached the ultimate shear strength. Local yielding or destruction occurred within the specimen at this stage, producing a small accumulation of plastic deformation. In the post-peak softening stage, the shear displacement reached a certain degree to form a shear damage surface, the shear stress gradually decreased, and the stress softening phenomenon appeared. However, brittle damage did not occur immediately because of the friction effect of the upper and lower shear surfaces and the residual shear strength provided by the cementation of the rock–slurry skeleton.

**Figure 12:**
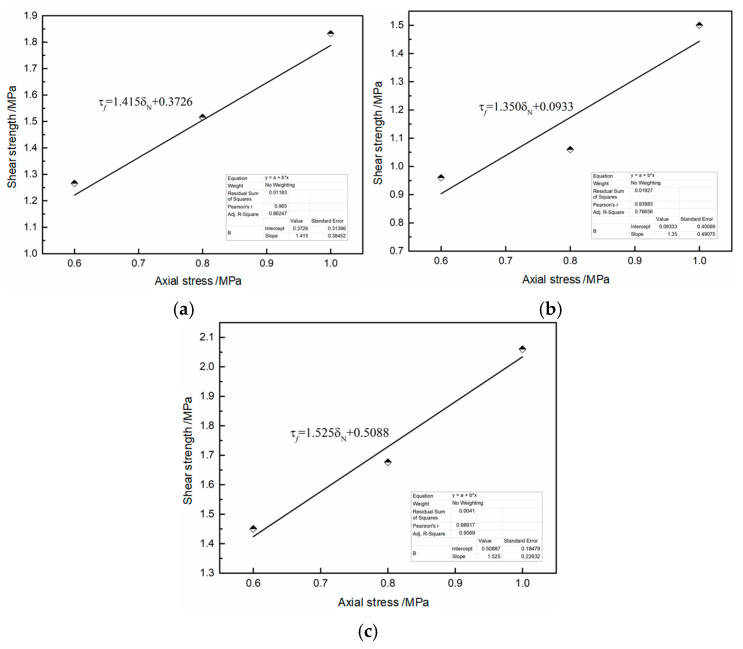
Relationship between shear strength and axial stress of SRM–SC: (**a**) Slurry A, rock content 40%. (**b**) Slurry B, rock content 40%. (**c**) Slurry C, rock content 40%.

The variation patterns of shear strength with axial stress for the SRM–SC specimens cemented with different types of slurries are plotted in Figure 12, and the data were linearly fitted to the shear strength of SRM–SC, as shown in Figure 12 using the Mohr–Coulomb strength criterion. It can be seen from Figure 12 that the shear strength of the SRM–SC specimens cemented with different types of slurry had a certain linear relationship with the axial stress, and the fitted straight line and data points had fitting degrees of 0.97, 0.94, and 0.99, respectively, which indicated that the Mohr–Coulomb strength criterion was applicable to the SRM–SC specimens formed by three types of slurry cementation in the range of axial stresses from 0.6 MPa to 1.0 MPa. Meanwhile, the corresponding parameters of the Mohr–Coulomb strength were obtained from the fitting results: the cohesive force, *c*, and the angle of internal friction, ϕ, and the Coulomb strength equations are summarized below:

(9) $$\tau_{f}=1.415\delta_{N}+0.3726,\;A–40\%$$

(10) $$\tau_{f}=1.350\delta_{N}+0.0933,\;B–40\%$$

(11) $$\tau_{f}=1.525\delta_{N}+0.5088,\;C–40\%$$

The cohesive force of the SRM–SC was mainly composed of the cementing force of the rock–slurry skeleton and the occlusal force between soil particles, rock particles, and slurry particles. According to the experimental fitting results, it can be seen that both the cohesive force and the angle of internal friction present the following pattern: SRM–SC cemented with ordinary Portland cement–water–glass slurry > SRM–SC cemented with ordinary Portland cement single slurry > SRM–SC cemented with low-alkalinity sulfoaluminate cement single slurry.

### 4.3. The Analysis of the Failure Characteristics of SRM–SC

#### 4.3.1. The Analysis of the Typical Failure Characteristics in Uniaxial Compression Tests

**Figure 13:**
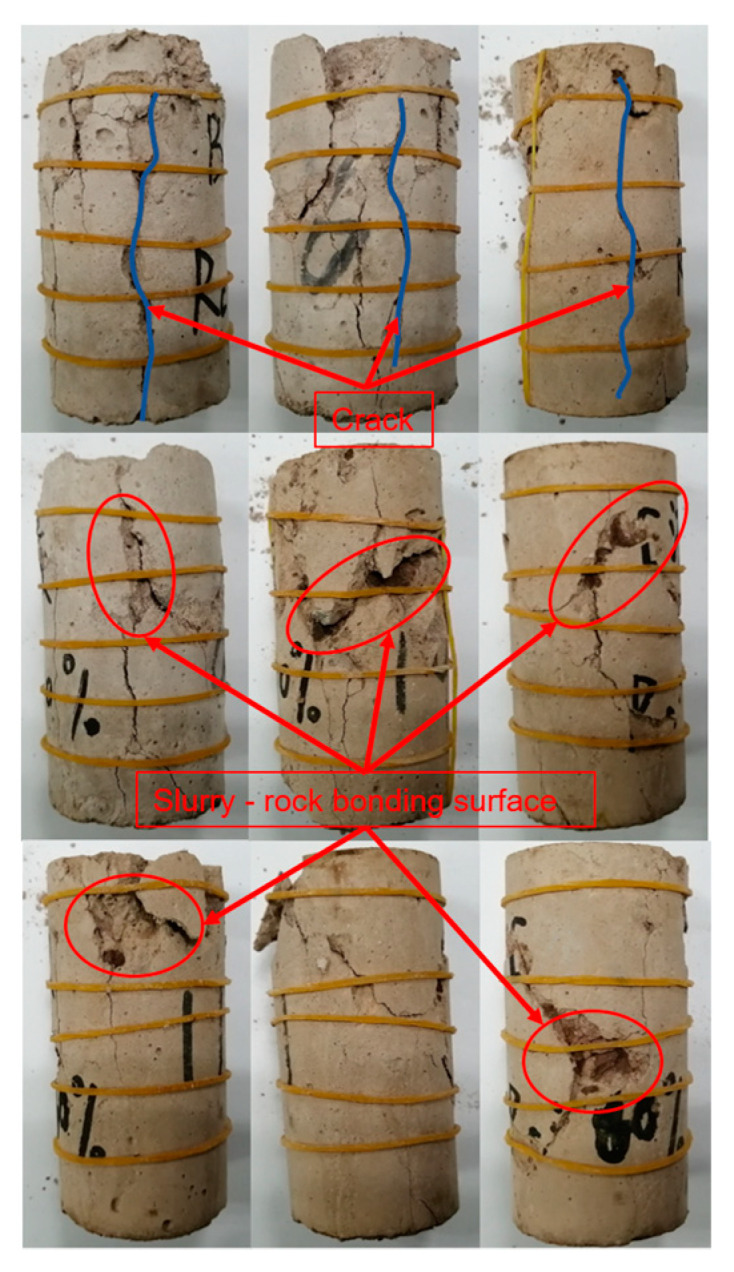
Typical failure form of the SRM–SC specimen under uniaxial compression.

According to Figure 13, the uniaxial compression damage of the SRM–SC specimen had the following typical features: 1. The predominant direction of cracks in the sample is vertical, with shear slip failure as the predominant failure mode. 2. The stress concentration damage occurred at the contact point between the indenter (steel ingot) and the top surface of the SRM–SC specimen, and there was a flaking phenomenon on the surface of the specimen. 3. Local damage occurred on the slurry–rock bonding surface.

#### 4.3.2. The Analysis of the Typical Failure Characteristics in Direct Shear Tests

**Figure 14:**
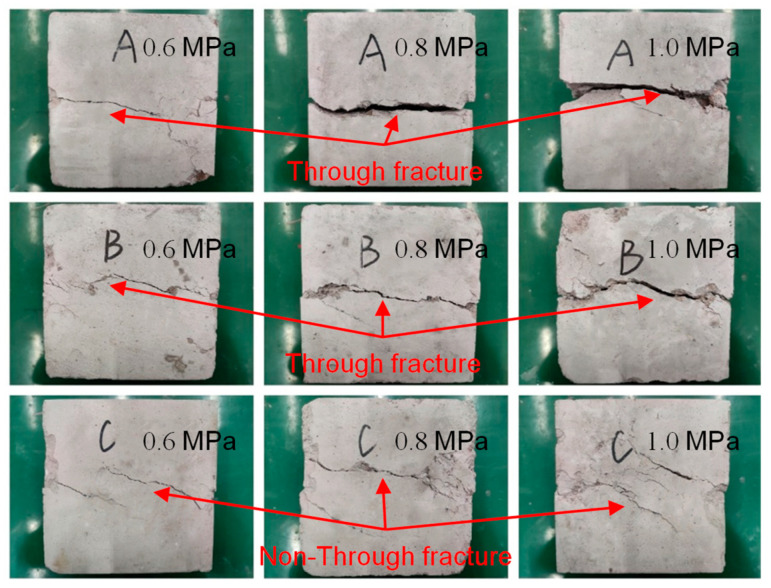
The shear failure form of the SRM–SC specimen surface.

**Figure 15:**
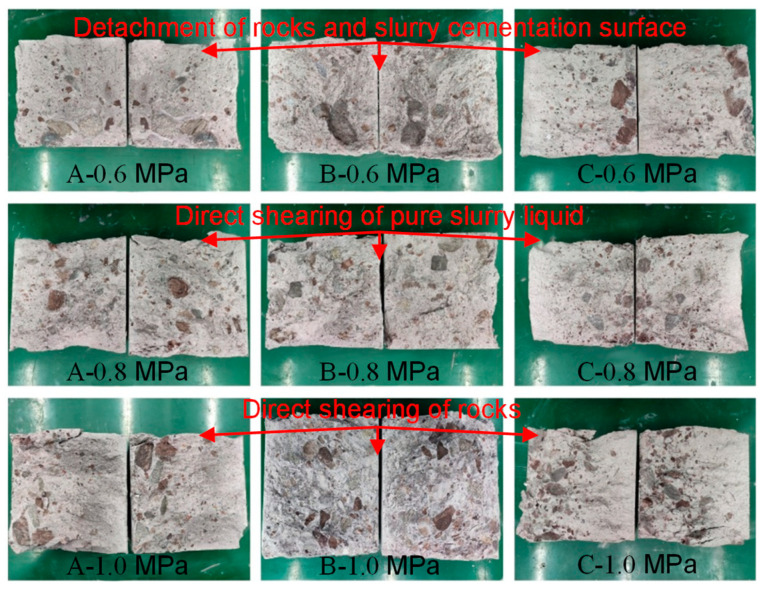
Shear surface characteristics of SRM–SC specimens.

According to Figure 14 and Figure 15, the direct shear damage of the SRM–SC specimens had the following typical characteristics:

1. The SRM–SC specimen cemented with ordinary Portland cement slurry (slurry A in Figure 14) and the SRM–SC specimen cemented with slurry (slurry B in Figure 14) produced a through fracture approximately parallel to the shear surface after reaching the peak shear strength; the fracture was dominated by the main fracture, with a lesser accompanying diagonal fracture. The SRM–SC specimen cemented with ordinary Portland cement–water–glass (slurry C in Figure 14) did not produce an apparent main fracture after reaching the peak shear strength, which indicated that its internal rock bite strength accounted for a more significant proportion than the slurry cement strength. In the soil–rock mixture, the internal rock bite strength of the rock refers to the resistance generated by the interlocking and meshing of rock particles. This bite action can enhance the overall strength and stability of the soil–rock mixture.
2. Shear surface damage was divided into three categories: the direct shearing of rocks, the detachment of rocks and the slurry cementation surface, and the direct shearing of hardened cement paste. The hardened cement paste does not contain any soil or rock fragments. The damage mode was related to the level of axial pressure. When the axial pressure level was larger, it was mainly to shear rocks and pure slurry liquid, and when the level of axial pressure was smaller, it was mainly the detachment of rocks and slurry cementation surface.
3. The main fracture shear surface with low flatness and a friction phenomenon was on the surface, which indicated that after the shear damage, the friction effect provides a certain residual strength.

## 5. Discussions

### 5.1. The Influence Regularity of Slurry Type on the Microstructure’s Property of SRM–SC

According to the electron microscope scanning test results of SRM–SC:

1. The weak cementing ability of the ordinary Portland cement slurry was determined by the inherent nature of the hydration reaction of ordinary Portland cement slurry, resulting in a small amount of calcium silicate hydrate and an uneven distribution. In contrast, the calcium silicate hydrate gel was the substance that played a fundamental role in the process of reinforcing the soil–rock mixture of the ordinary Portland cement slurry.
2. The hydration reaction of the low-alkalinity sulfoaluminate cement slurry was sufficient to produce a large amount of ettringite and calcium silicate hydrate to fill the void between the covering soil particles and rock particles and establish strong cementation between the soil–rock mixture particles and particles, as well as between the particle surfaces and the slurry, to give it a certain degree of structural strength. In the early stage of hydration, the ettringite reaction was generated quickly because ettringite has the role of counteracting contraction and generating mechanical prestress, so it first plays the role of gelling and then the generation of calcium silicate hydrate to form a cemented skeleton; after the completion of hydration, ettringite also has an expansion effect. As mentioned above, from the analysis of the cementing ability, only the cementing ability of the low-alkalinity sulfoaluminate cement slurry was powerful. Ettringite was the main component of the low-alkalinity sulfoaluminate cement slurry in the process of reinforcing the soil–rock mixture, which not only has a cementing effect but also, through its expansion characteristics, compacts further the bulk structure of the soil–rock mixture.
3. The microstructures of the ordinary Portland cement–water–glass slurry cementation and the ordinary Portland cement slurry cementation are generally the same, and the difference lies in the amount of calcium silicate hydrate generated, which also leads to the difference in the effect of calcium silicate hydrate on the filling of the gap cementation between the particles of the soil–rock mixture.

Comprehensively comparing the microstructures of the three types of slurry cement from the view of the cement filling capacity, we identified that the SRM–SC formed by using different types of slurry reinforcement in the microscopic cement had apparent differences and present the following law: low-alkalinity sulfoaluminate cement slurry > ordinary Portland cement–water–glass slurry > ordinary Portland cement slurry. Therefore, it was necessary to choose the corresponding grouting materials according to the different needs and characteristics of grouting projects and different stratum conditions. Among the above three grouting materials, the low-alkalinity sulfoaluminate cement slurry had an optimal filling effect.

### 5.2. The Influence Regularity of the Slurry Type on the Compressive Properties of SRM–SC

**Figure 16:**
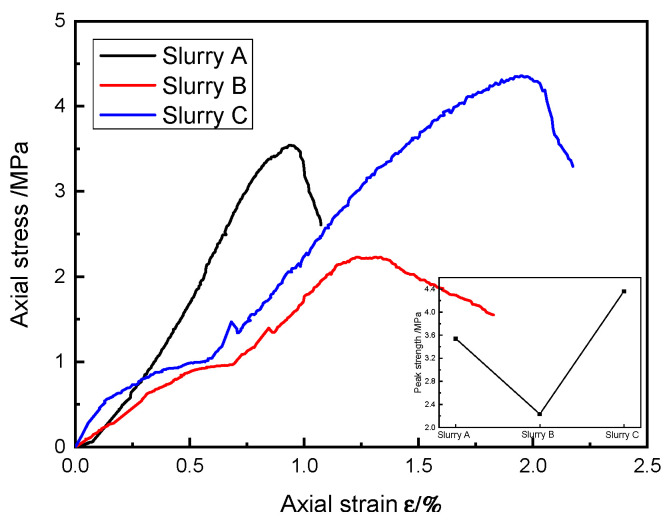
The influence of the slurry type on the compressive strength of SRM–SC (rock content 40%).

The stress–strain curves of the typical specimens of SRM–SC under uniaxial compression conditions for each group of different cementitious materials are shown in Figure 16, with rock contents of 40%.

The SRM–SC was a discontinuous composite material, internally composed of four phases: soil, rock, slurry, and voids. Its stress–strain relationship curve was similar to that of ordinary concrete materials, which was divided into three stages: the compression–density stage, the approximate elasticity stage, and the destruction stage. As shown in Figure 16, at the beginning of the test that began the compression stage, the original small void in the SRM–SC specimen was compressed, and there was a gradual increase in the slope of the axial stress–strain curve and the compressive deformation capacity. The axial strain increased to a certain extent into the approximate elasticity of the stage. Due to the discontinuity of the material, the axial stress and axial strain as a whole presented a linear elasticity of the relationship between the tangent lines of the local slopes on each point, and there was a sudden change. When the ultimate load of the SRM–SC specimen entered the destructive stage, the initial crack expanded rapidly under the action of the sharp release of elastic energy, i.e., brittle damage occurred, and the complete stress–strain curve could not be measured accurately. Based on the compressive mechanical behavior of the SRM–SC, it can be classified as a brittle material, which will eventually be crushed when subjected to compression, but the compressive strength limit was much greater than the tensile strength limit.

**Figure 17:**
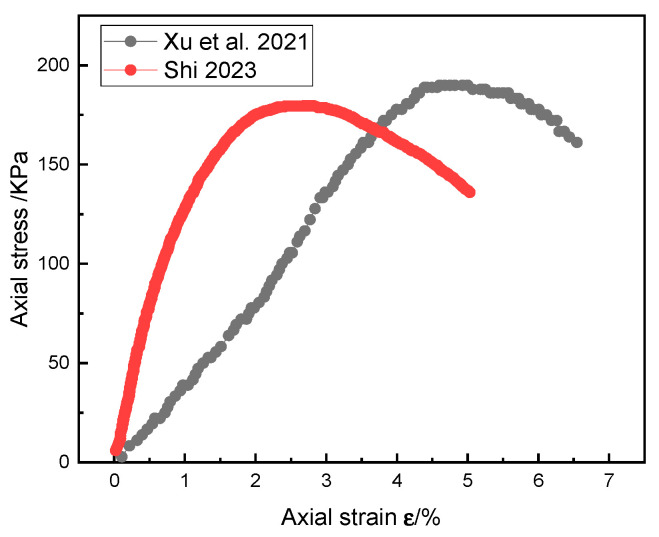
The axial stress–strain curve of the soil–rock mixture (rock content 40%) [34,35].

Upon comparing Figure 16 and Figure 17, it is evident that after reaching the peak axial stress, the axial stress of SRM–SC decreases at a significantly higher rate with the increasing axial strain compared to that of the soil–rock mixture. This indicates that the soil–rock mixture tends to exhibit plastic failure, whereas the SRM-SC is more prone to brittle failure.

Under the condition of the same rock content of the SRM–SC specimens, the uniaxial compressive ultimate strength of the SRM–SC bonded with the ordinary Portland cement–water–glass slurry was the highest, followed by the SRM–SC specimens cemented with the ordinary Portland cement slurry, and the lowest was the SRM–SC specimens cemented with the low-alkalinity sulfoaluminate cement slurry. The uniaxial compressive ultimate strengths of the SRM–SC specimens formed by the three kinds of slurry cement were 3.54 MPa, 2.23 MPa, and 4.36 MPa at the 40% rock content.

**Figure 18:**
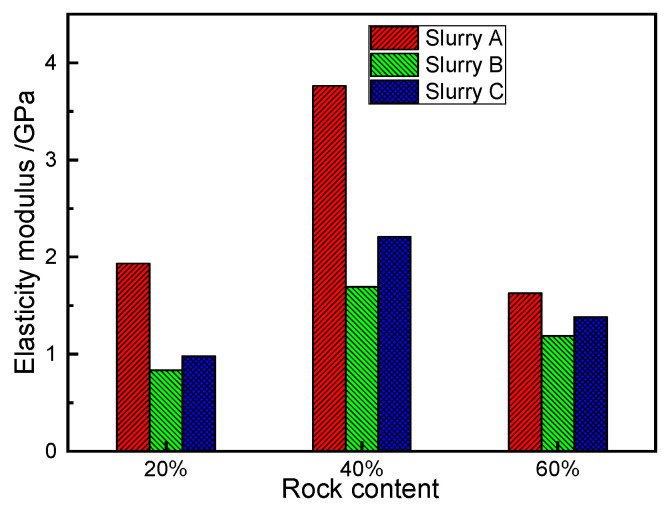
The comparison of the elasticity modulus of the SRM–SC under different slurry types.

A comparison of the elasticity modulus of the SRM–SC under the different types of slurry cementing conditions is shown in Figure 18, which shows that the elasticity moduli of the SRM–SC specimens cemented with the ordinary Portland cement slurry was the largest under the conditions of each rock content: 1.93 GPa, 3.76 GPa, and 1.62 GPa, respectively; the SRM–SC specimens cemented with the ordinary Portland cement–water–glass slurry were the second largest: 0.83 GPa, 1.69 GPa, and 1.18 GPa, respectively; and the SRM–SC specimens cemented with the low-alkalinity sulfoaluminate cement slurry were the smallest: 0.98 GPa, 2.21 GPa and 1.38 GPa, respectively.

In summary, the type of slurry is an essential factor affecting the compressive mechanical properties of the SRM–SC. Among them, the SRM–SC cemented with the ordinary Portland cement–water–glass slurry had the strongest ability to resist damage.

### 5.3. The Influence Regularity of the Rock Content on the Compressive Properties of SRM–SC

**Figure 19:**
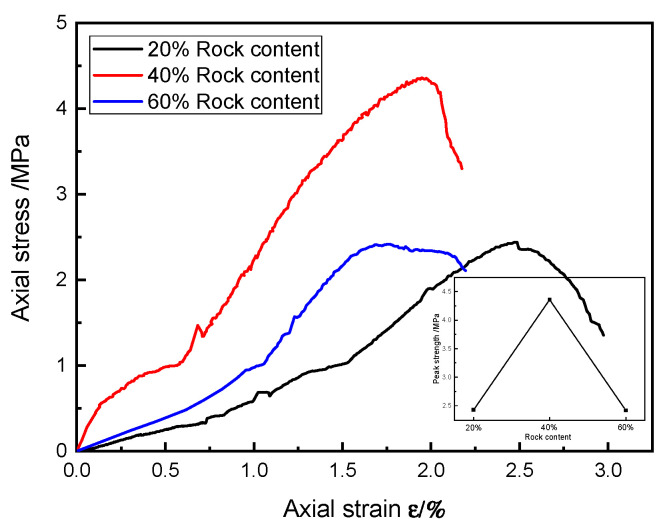
The influence of the rock content on the compressive strength of SRM–SC (slurry C).

**Figure 20:**
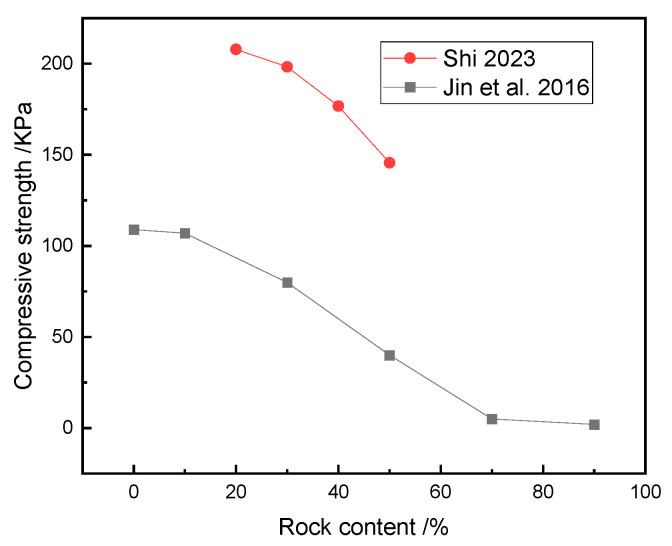
The influence of the rock content on the compressive strength of the soil–rock mixture [35,36].

The stress–strain curves of the typical specimens of SRM–SC with different rock contents under uniaxial compression are shown in Figure 19. As seen from Figure 19, under the condition of using the same type of slurry cement, the SRM–SC with the 40% rock content had the highest uniaxial compressive ultimate strength, and the uniaxial compressive ultimate strengths of the SRM–SC specimens with the 20% rock content and the 60% rock content were relatively close to each other. The uniaxial compressive ultimate strength of the SRM–SC samples cemented with the ordinary Portland cement–water–glass slurry was 2.43 MPa, 4.36 MPa, and 2.42 MPa, respectively. It is evident that the uniaxial compressive ultimate strength of the SRM–SC exhibits a trend of initial enhancement followed by a decline with the increasing rock content, identifying an inflection point between the 20% and the 60% rock content. As depicted in Figure 20, the uniaxial compressive ultimate strength of the soil–rock mixture decreases monotonically with the rise in the rock content. This indicates that the grouting reinforcement induces alterations in the internal microstructure of the soil–rock mixture, consequently modifying its uniaxial compressive behavior.

**Figure 21:**
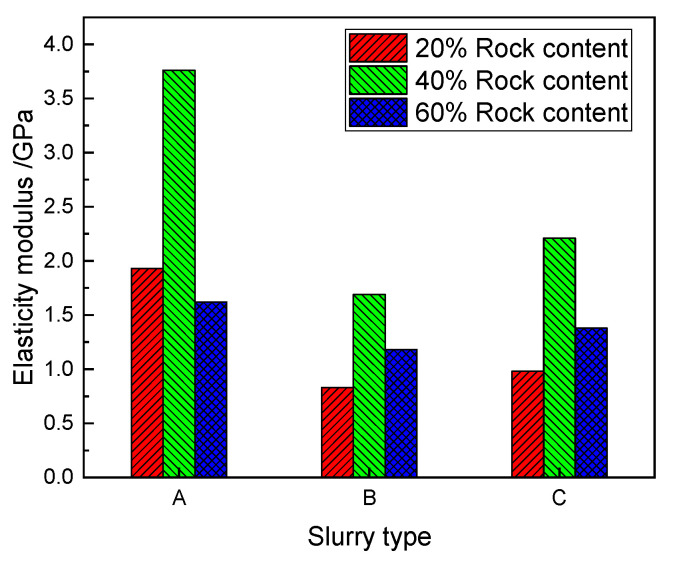
Comparison of elastic moduli of SRM–SC under different rock content conditions.

A comparison of the elasticity modulus of the SRM–SC under the different rock content conditions is shown in Figure 21: under the condition of using the same type of slurry cement, the elasticity moduli of the SRM–SC specimens with the 40% rock content were the highest, which were 3.76 GPa, 1.69 GPa, and 2.21 GPa, respectively; the elasticity moduli of the SRM–SC specimens with the 20% rock content were 1.93 GPa, 0.83 GPa, and 0.98 GPa; the elasticity moduli of the SRM–SC specimens with the 60% rock content were 1.62 GPa, 1.18 GPa, and 1.38 GPa, respectively.

In summary, the rock content was also an important factor affecting the compressive mechanical properties of the SRM–SC. Using the same type of slurry and grouting parameters for formation reinforcement in grouting areas with different rock contents would produce different reinforcement effects. Therefore, it was necessary to adopt a targeted grouting reinforcement program according to the index of the rock content of the strata in the geotechnical investigation report of the interval. Among them, the SRM–SC with the 40% rock content had the strongest ability to resist both damage and deformation. This was because the rock–slurry skeleton and the cemented soil mainly determined the compressive strength of the SRM–SC.

When the rock content was low, the rock distribution was sparse and could not effectively form a rock–slurry skeleton to play a supporting role, and the cemented soil mass dominated the overall strength. In contrast, the compressive capacity of the separate cemented soil mass was limited, its destruction was regarded as the overall destruction, and the strength of the rock was not utilized, resulting in the low strength of the specimens with low rock contents. When the rock content was high, in the case of a certain slurry binder, too much rock would lead to the formation of an insufficient rock–slurry skeleton bonding capacity, and the skeleton contact surface became an overall structurally weak surface. At the same time, the continuity of the cemented soil mass was reduced, and fractures easily developed along the established weak surface of the damage, resulting in the low strength of the specimens with high rock contents. When the rock content was moderate, it could not only meet the cementation capacity of the rock–slurry skeleton to form a support but also fully utilized the continuous filling effect of the cemented soil mass, so the strength was higher than that of the other rock content conditions. The test data also indicate an inflection point for the SRM–SC to change from a strong to a weak strength at between aa 20% and a 60% rock content.

### 5.4. The Influence Regularity of the Slurry Type on the Shear Properties of SRM–SC

The shear stress–shear displacement curves of the typical specimens of SRM–SC reinforced with different types of slurries are shown in Figure 11. The data in Figure 11 show that the corresponding shear strengths were 1.27 MPa, 0.96 MPa, and 1.45 MPa under the cementation conditions of slurry A, slurry B and slurry C, and the peak shear displacements were 2.08 mm, 2.76 mm, and 1.64 mm, respectively, and the corresponding shear moduli were 0.61 GPa, 0.35 GPa, and 0.88 GPa, respectively.

Comprehensively, it can be seen that there were significant differences in the macroscopic mechanical properties of the SRM–SC reinforced by different types of slurries. For the three grouting materials with the most extensive engineering applicability, the reinforcement effect of ordinary Portland cement–water–glass slurry had better performance in compressive strength, shear strength, and resistance to corresponding deformation.

### 5.5. The Influence Regularity of the Axial Pressure on the Shear Properties of SRM–SC

**Figure 22:**
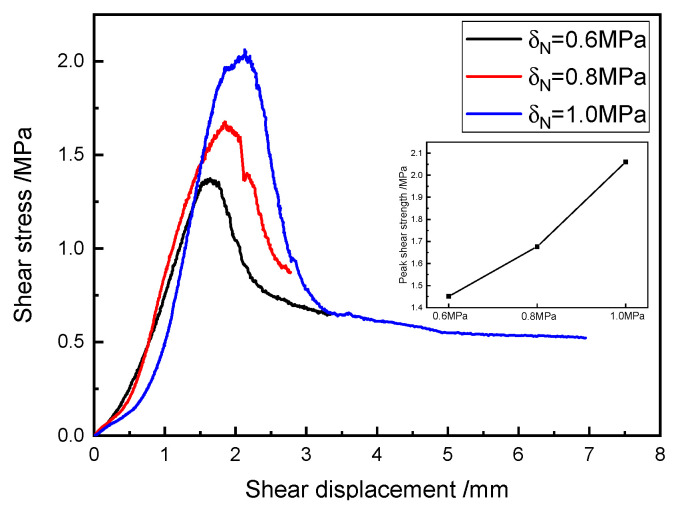
The influence of axial pressure on the shear resistance of SRM–SC (slurry C, rock content 40%).

The shear stress–shear displacement curves of the typical specimens of SRM–SC under different axial pressure conditions are shown in Figure 22. The data in Figure 22 show that the corresponding shear strengths were 1.45 MPa, 1.68 MPa, and 2.06 MPa for axial pressures of 0.6 MPa, 0.8 MPa, and 1.0 MPa, respectively, the peak shear displacements were 1.64 mm, 1.86 mm, and 2.13 mm, respectively, and the corresponding shear moduli were 0.88 GPa, 0.90 GPa, and 0.97 GPa, respectively.

It can be concluded that the axial pressure was an important factor affecting the shear mechanical properties of the SRM–SC. With the increase in the axial pressure, the shear strength and the shear stiffness of the SRM–SC also increased. As mentioned earlier, the relationship between the shear strength of the SRM–SC and the axial stress was verified to align with the Mohr–Coulomb criterion. That is, increasing the axial pressure can enhance the ability of SRM–SC to resist deformation and damage, and the reasons for this were analyzed as follows: on the one hand, the axial pressure can play a role in compacting the original tiny voids and fractures within the range of not exceeding the uniaxial compressive strength of the SRM–SC specimen, which improves the continuity of the internal structure of the SRM–SC specimen; on the other hand, increasing the axial stress can enhance the friction effect between the particles inside the SRM–SC specimen, and the maximum static friction at the shear position increases, at which time it is necessary to provide larger shear stress to overcome the frictional resistance, thus indirectly enhancing the shear strength and shear modulus of the SRM–SC.

### 5.6. The Influence Regularity of the Rock Content on the Shear Properties of SRM–SC

**Figure 23:**
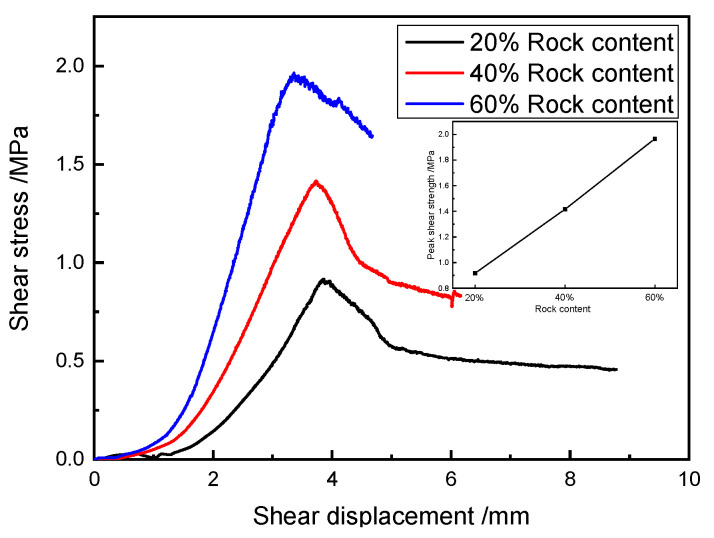
The influence of the rock content on the shear resistance of SRM–SC (slurry A, axial pressure $\delta_{N}=0.8\;MPa$).

The shear stress–shear displacement curves of the typical specimens of SRM–SC with different rock contents under direct shear conditions are shown in Figure 23. The data in Figure 23 show that the corresponding shear strengths of the SRM–SC specimens with 20%, 40%, and 60% rock contents were 0.92 MPa, 1.41 MPa, and 1.96 MPa, respectively, the peak shear displacements were 3.86 mm, 3.75 mm, and 3.36 mm, respectively, and the corresponding shear moduli were 0.24 GPa, 0.38 GPa, and 0.58 GPa, respectively.

**Figure 24:**
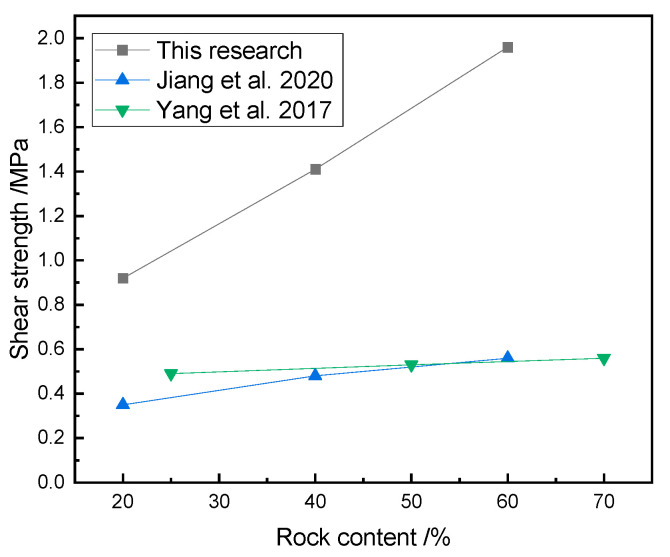
The influence of the rock content on the compressive strength of the soil–rock mixture/SRM–SC (axial pressure $\delta_{N}=0.8\;MPa$) [37,38].

Obviously, the rock content was an essential factor affecting the shear mechanical properties of the SRM–SC, and the shear strength and shear stiffness of the SRM–SC increased with the increasing rock content. The increased rock content contributes to the formation of a slurry–rock bonded framework on the shear plane and a higher density of rock particles, thereby elevating the resistance that must be overcome to induce shearing. On the other hand, with the increased rock content, the slurry–rock skeleton and the contact surface of the cemented soil mass increased, and the coupling and interlocking effect between the rocks and between the rocks and the slurry was also enhanced. This regularity is in agreement with the investigation into the shear behavior of soil–rock mixtures (refer to Figure 24), as the shear strength of rock–soil mixtures and SRM–SC both increase with the increase in rock content. The distinct difference lies in the fact that the rate of increase in the shear strength of the SRM–SC with the increasing rock content is significantly greater than that of the soil–rock mixtures. This suggests that the shear strength of the SRM–SC is more sensitive to changes in the rock content compared to that of the soil–rock mixtures.

**Figure 25:**
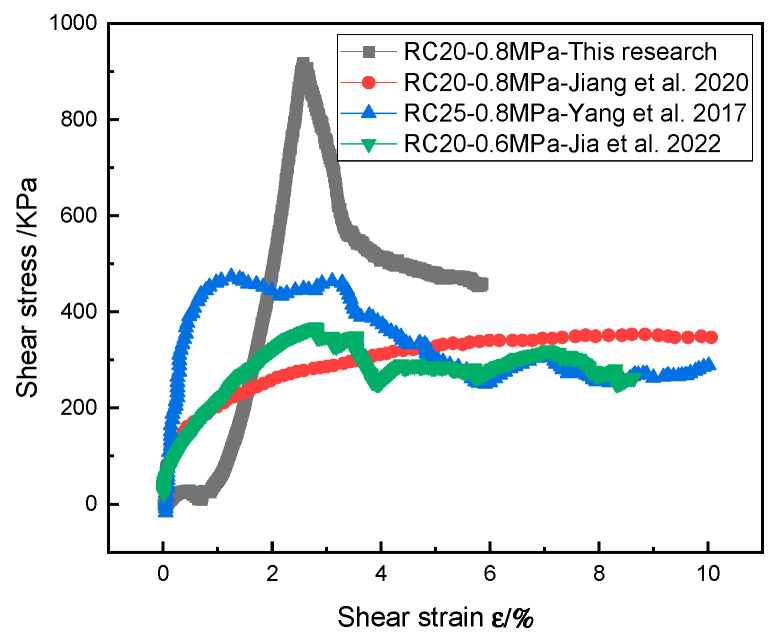
The shear stress–strain curve of the soil–rock mixture/SRM–SC (note: RC20-0.8 MPa represents a rock content of 20% and an axial pressure of 0.8 MPa) [37,38,39].

Figure 25 illustrates that the shear stress–shear strain curve for the soil–rock mixture can be categorized into three distinct phases. The initial phase is characterized as the shear densification stage, wherein shear stress rises swiftly, and the curve presents a notable convex profile. The second phase is identified as the elastic deformation stage, where the curve manifests a near-linear ascending trend. The final phase is the plastic strain stage, during which the rate of the shear stress incremental increases progressively declines, leading to a reduction in the curve’s slope. Additionally, this stage may exhibit a decrease in the shear stress with the increasing shear strain, a phenomenon known as strain softening. Furthermore, the shear stress–shear strain curve for the SRM–SC, as depicted in Figure 25, reveals a sharp decline in the shear stress following the peak. This is consistent with the brittle nature of the soil–rock mixture after the grouting process.

Combined macromechanical property testing and microproperty testing of the SRM–SC were performed, both of which formed a mutual validation:

1. Macroscopically, the SRM–SC specimen cemented with the ordinary Portland cement–water–glass slurry had advantages in mechanical properties; microscopically, it presented fewer defects on the cementing surface, a more uniform and dense distribution of calcium silicate hydrate, and an apparent cementing effect. This was because adding a water–glass aqueous solution accelerated the ordinary Portland cement’s hydration reaction and generated more calcium silicate hydrate cementation products, high early strength, and late strength. The shortcomings were that the impurities in the water–glass aqueous solution caused the poor durability of the SRM–SC.
2. Macroscopically, the SRM–SC specimen cemented with the low-alkalinity sulfoaluminate cement slurry had the characteristics of high strength in the early stage and insufficient strength in the late stage; microscopically, it showed that ettringite fills and covers the whole cement surface, and the structural compactness was obviously improved compared with the SRM–SC cemented with the other two types of slurry because of the fast rate of the reaction of the low-alkalinity sulfoaluminate cement to generate ettringite, and its quantity was high. The ettringite crystals interlaced with each other between the particles of the soil–rock mixture to form the backbone, rapidly forming the early strength. However, due to the expansion characteristics of ettringite, the internal stress exceeded the tensile strength of the cement and produced minor fractures in the late stage, which manifested as a loss of strength and a reduction in the elastic modulus at the macroscopic level.
3. The SRM–SC specimen cemented with the ordinary Portland cement slurry had higher late strength than the SRM–SC specimen cemented with the low-alkalinity sulfoaluminate cement slurry in terms of macroscopic mechanical properties but had the worst microscopic cementation properties.

## 6. Conclusions

This study analyzed different slurries’ cementing ability and mechanisms using scanning electron microscopy observations. Then, through laboratory uniaxial compression tests and direct shear tests, the macromechanical properties of SRM–SC were studied, considering factors such as the rock content, slurry type, and axial stress level, and macromicroscopic comparisons and verifications were performed. The main conclusions are summarized as follows:

(1) The rock content significantly affects the macromechanical properties of an SRM–SC; the compressive strength and stiffness of the SRM–SC is stronger and then weaker with the increasing rock content. For instance, utilizing slurry A as a representative, the compressive strengths recorded at rock contents of 20%, 40%, and 60% were 1.88 MPa, 3.54 MPa, and 1.85 MPa, respectively. Correspondingly, the elastic moduli measured were 1.93 GPa, 3.76 GPa, and 1.62 GPa, respectively. Notably, an inflection point was observed within the range of the rock content being between 20% and 60%. The shear strength and stiffness both increased with the increasing rock content. Taking slurry A as a representative, the recorded shear strengths at rock contents of 20%, 40%, and 60% were 0.92 MPa, 1.41 MPa, and 1.96 MPa, respectively. Correspondingly, the shear moduli measured were 0.24 GPa, 0.38 GPa, and 0.58 GPa, respectively.
(2) The macroscopic mechanical properties of SRM–SCs formed by different types of slurry reinforcement differed significantly. Among them, the SRM–SC specimen cemented with the ordinary Portland cement–water–glass slurry had the best compressive, shear, and deformation resistance performance, the SRM–SC specimen cemented with the ordinary Portland cement slurry was the second best, and the SRM–SC specimen cemented with the low-alkalinity sulfoaluminate cement slurry was poor. The addition of water–glass can obviously shorten the initial setting time of the slurry and quickly provide early strength, and the later strength can be significantly improved. It is recommended to use an ordinary Portland cement–water–glass slurry as the grouting reinforcement material in engineering applications.
(3) The SRM–SC basically conforms to the Mohr–Coulomb criterion in the range of axial stress 0.6 MPa~1.0 MPa. The damage mode of the shear surface was divided into three categories: the direct shearing of rock, the detachment of the rock and slurry cementation surface, and the direct shearing of pure slurry liquid. The damage mode is related to the axial pressure level; when the axial pressure level is higher, it is dominated by the shearing of the rock and pure slurry liquid, and when the axial pressure level is less, it is dominated by the detachment of the rock and slurry cementation surface.
(4) The microscopic cementation mechanism of SRM–SC is related to the hydration products produced by the hydration reaction of different types of slurries, and the formation quantity, formation speed, and characteristics of the hydration products directly determine the strengthening and cementation effect. The hydration products are represented by calcium silicate hydrate and ettringite.

## Figures and Tables

**Table 1:** Fundamental performance indicators of ordinary Portland cement.

Specific Surface Area $(m^{2}/kg$)	Setting Time (min)		Flexural Strength (MPa)		Compressive Strength (MPa)		Content of Supplementary Cementitious Material/Filler (%)		Gypsum (%)
	Initial Setting Time	Final Setting Time	3 d	28 d	3 d	28 d	slag	Calcium Carbonate	
342	163	223	6.1	8.6	32.2	50.8	2.29	7.42	5

**Table 2:** Fundamental performance indexes of low-alkalinity sulfoaluminate cement.

Specific Surface Area $(m^{2}/kg$)	Setting Time (min)		Flexural Strength 1 d (MPa)	Compressive Strength 1 d (MPa)	pH	Calcium Carbonate (%)	28 d Free Swelling Ratio (%)
	Initial Setting Time	Final Setting Time					
473	10	15	6.2	37.5	9.6	19	0.07

**Table 3:** Fundamental performance indicators of water–glass.

Density 20 °C $(g/cm^{3}$)	$Na_{2}O$ (%)	$SiO_{2}$ (%)	Modulus (M)	Baume Degree $(Be^{\prime }$)
1.378	8.49	26.85	3.26	40°

**Table 4:** Fundamental physical parameters of the soil–rock mixture samples.

Silty Clay				Argillaceous Sandstone			
Natural Moisture Content (%)	Soil Natural Density $(g/cm^{3}$)	Specific Gravity	Natural Void Ratio	Natural Density $(g/cm^{3}$)	Saturated Density $(g/cm^{3}$)	Compressive Strength (MPa)	Elastic Modulus (GPa)
21.6	2.02	2.71	0.63	2.49	2.52	27.35	3.43

**Table 5:** The test scheme for the direct shear of SRM–SC.

Sample Number	Rock Content (%)	Slurry Type	Axial Pressure (MPa)	Soil:Rock:Slurry Ratio
1	40	A	0.6	3:2:2
2		B		
3		C		
4	40	A	0.8	3:2:2
5		B		
6		C		
7	40	A	1.0	3:2:2
8		B		
9		C		
10	20	A	0.8	4:1:2
11	60			2:3:2

**Table 6:** Test scheme for uniaxial compression of SRM–SC.

Sample Number	Rock Content (%)	Slurry Type	Soil:Rock:Slurry Ratio
a	20	A	4:1:2
b		B	
c		C	
d	40	A	3:2:2
e		B	
f		C	
g	60	A	2:3:2
h		B	
i		C	

**Table 7:** Compressive strength and corresponding peak strain of SRM–SC.

Slurry Type	Rock Content (20%)		Rock Content (40%)		Rock Content (60%)	
	Compressive Strength (MPa)	Strain (%)	Compressive Strength (MPa)	Strain (%)	Compressive Strength (MPa)	Strain (%)
A	1.88	0.972	3.54	0.941	1.85	1.137
B	1.77	2.124	2.23	1.317	1.53	1.289
C	2.43	2.476	4.36	1.974	2.42	1.751

**Table 8:** Shear strength and corresponding peak shear displacement of SRM–SC.

Rock Content (%)	Arial Compressive Force (MPa)	Slurry Type	Shear Strength (MPa)	Peak Shear Displacement (mm)
40	0.6	A	1.27	2.08
		B	0.96	2.76
		C	1.45	1.64
	0.8	A	1.41	3.75
		B	1.06	3.39
		C	1.68	1.86
	1.0	A	1.83	2.77
		B	1.50	2.70
		C	2.06	2.13
20	0.8	A	0.92	3.86
60	0.8	A	1.96	3.36

## Data Availability

The data presented in this study are available in this article. Further inquiries can be directed to the corresponding author.

## References

[1] Wang Y., Que J.M., Wang C., Li C.H. (2018). Three-dimensional observations of meso-structural changes in bimsoil using X-ray computed tomography (CT) under triaxial compression. Constr. Build. Mater..

[2] Zhang Z.L., Xu W.J., Xia W., Zhang H.Y. (2016). Large-scale in situ test for mechanical characterization of soil-rock mixture used in an embankment dam. Int. J. Rock Mech. Min. Sci..

[3] Zhang H.Y., Xu W.J., Yu Y.Z. (2016). Numerical analysis of soil-rock mixture’s meso-mechanics based on biaxial test. J. Cent South. Univ..

[4] Chang C.S., Meidani M., Deng Y. (2017). A compression model for sand-silt mixtures based on the concept of active and inactive voids. Acta. Geotech..

[5] Li Z.Q., Hu F., Qi S.W., Hu R.L. (2020). Strain-softening failure mode after the post-peak as a unique mechanism of ruptures in a frozen soil-rock mixture. Eng. Geol..

[6] Moufida E.M., Dhekra S., Hela B.S., Mahmoud D. (2015). Geotechnical characterization of the quaternary alluvial deposits in Tunis City (Tunisia). J. Afr. Earth. Sci..

[7] Chen L., Yang Y.T., Zheng H. (2018). Numerical study of soil-rock mixture: Generation of random aggregate structure. Sci. Chin. Technol. Sci..

[8] Xu W.J., Wang S., Zhang H.Y., Zhang H.Y. (2016). Discrete element modelling of a soil-rock mixture used in an embankment dam. Int. J. Rock Mech. Min. Sci..

[9] Zhong Z.L., Li J.Y., Bie C.Y. (2023). Theoretical Approach to Predicting the Diffusion Radius of Fracture Grouting in Soil–Rock Mixtures. Appl. Sci..

[10] Avsar E. (2021). An experimental investigation of shear strength behavior of a welded bimrock by meso-scale direct shear tests. Eng. Geol..

[11] Wang Y., Li X., Zheng B., Mao T.Q., Hu R.L. (2016). Investigation of the effect of soil matrix on flow characteristics for soil and rock mixture. Geotech. Lett..

[12] Dan H.C., He L.H., Xu B. (2016). Experimental Investigation on Non-Darcian Flow in Unbound Graded Aggregate Material of Highway Pavement. Transport. Porous. Med..

[13] Wang Y., Li X., Zheng B., Zhang Y.X., Li G.F., Wu Y.F. (2016). Experimental study on the non-Darcy flow characteristics of soil–rock mixture. Environ. Earth. Sci..

[14] Zhong Z.L., Zou H., Hu X.X., Liu X.R. (2021). Experimental study on stiffness softening of soil–rock mixture backfill under metro train cyclic load. Adv. Mater. Sci. Eng..

[15] Huang F., Mi J.L., Yang Y.H., Dong G.F., Zhang B., Liu X.C. (2024). Morphological characteristics of hysteretic curves of soil–rock mixture under stepped axial cyclic loading. Rock. Soil. Mech..

[16] Wang P.S., Hu W.J., Liu P.Y., Yan Z.Q., Kong X.H., Zhao Q.M., Yin W.H. (2022). An Experimental Study on Dynamic Characteristics of Coarse-Grained Soil Under Step Cyclic Loading. Coatings.

[17] Tian Y.C., Liu Q.S., Ma H., Liu Q., Deng P.H. (2018). New peak shear strength model for cement filled rock joints. Eng. Geol..

[18] Zhang Z.P., Sheng Q., Fu X.D., Zhou Y.Q., Huang J.H., Du Y.X. (2020). An approach to predicting the shear strength of soil–rock mixture based on rock block proportion. Bull. Eng. Geol. Environ..

[19] Wang Y., Feng W.K., Li C.H., Hou Z.Q. (2020). An investigation into the effects of block size on the mechanical behaviors of bimsoils using variable-angle shear experiments. Environ. Earth. Sci..

[20] Qian J.F., Yao Y.S., Li J., Xiao H.B., Lou S.P. (2020). Resilient Properties of Soil–Rock Mixture Materials: Preliminary Investigation of the Effect of Composition and Structure. Materials.

[21] Jin L., Zeng Y.W., Xia L., Ye Y. (2017). Experimental and Numerical Investigation of Mechanical Behaviors of Cemented Soil–Rock Mixture. Geotech. Geol. Eng..

[22] Ding X.H., Shi X.Y., Zhou W., Luan B.Y. (2019). Experimental Study on the Permeability of a Soil–Rock Mixture Based on the Threshold Control Method. Adv. Civ. Eng..

[23] Park K., Kim D. (2016). Analysis of Homogel Uniaxial Compression Strength on Bio Grouting Material. Materials.

[24] Shi Z., Wang Q., Xu L. (2020). Experimental Study of Cement Alkali-Resistant Glass Fiber (C-ARGF) Grouting Material. Materials.

[25] Wu Y.H., Li Q.Q., Li G.X., Tang S.Y., Niu M.D., Wu Y.F. (2021). Effect of Naphthalene-Based Superplasticizer and Polycarboxylic Acid Superplasticizer on the Properties of Sulfoaluminate Cement. Materials.

[26] Ma H., Liu Q. (2017). Prediction of the Peak Shear Strength of Sandstone and Mudstone Joints Infilled with High Water-Cement Ratio Grouts. Rock Mech. Rock Eng..

[27] Tian H.M., Chen W.Z., Yang D.S., Yang J.P. (2015). Experimental and Numerical Analysis of the Shear Behavior of Cemented Concrete-Rock Joints. Rock Mech. Rock Eng..

[28] Zhang H., Hu X., Boldini D., He C.C., Liu C., Ai C.J. (2020). Evaluation of the shear strength parameters of a compacted S-RM fill using improved 2-D and 3-D limit equilibrium methods. Eng. Geol..

[29] Mahdevari S., Moarefvand P., Mohammadzamani D. (2020). Considering the Effect of Block-to-Matrix Strength Ratio on Geomechanical Parameters of Bimrocks. Geotech. Geol. Eng..

[30] Koohestani B., Belem T., Koubaa A., Bussiere B. (2016). Experimental investigation into the compressive strength development of cemented paste backfill containing Nanosilica. Cement. Concrete. Comp..

[31] Shaikh F.U.A., Shafaei Y., Sarker P.K. (2016). Effect of nano and micro-silica on bond behavior of steel and polypropylene fibers in high volume fly ash mortar. Constr. Build. Mater..

[32] (2019). Test Preparation of Coarse Granular Soil. Standard for Geotechnical Tesing Method.

[33] Zhong Z.L., Tu Y.L., He X.Y., Feng J.H., Wang Z.L. (2016). Research Progress on Physical Index and Strength Characteristics of Bimsoils. Chin. J. Undergr. Sp. Eng..

[34] Xu H., Zhou T.Y., Wang X.Y., Zhang J., Zhang X.B., Liu Y.C. (2021). Compression test and numerical simulation research on improved red beds subgrade fillers in Sichuan-Tibet Railway. Rock. Soil. Mech..

[35] Shi S.Z. (2023). Experimental Study on the Mechanical and Acoustic Characteristics of Soil Rock Mixture. Master’s Thesis.

[36] Jin L., Zeng Y.W., Ye Y. (2016). Three–Dimensional Particle Flow Simulation of Uniaxial Compression Tests on Soil–rock Mixture. J. Changjiang River. Sci. Res. Inst..

[37] Jiang Q.Q., Xu Y.Q., Wang H. (2020). Research on shear deformation characteristics of soil–rock mixtures under different stone contents. J. Eng. Geol..

[38] Yang Z.P., Lei X.D., Wang L., Hu Y.X., Liu Y.Q. (2017). Impact of stone content to shear properties of soil–rock mixture using particle flow code simulation. J. Eng. Geol..

[39] Jia C.J., Liang G.D., Huang J., Lei M.F., Zhao C.Y., Zhang Q., Zhang J. (2022). Study on mechanical characteristics of soil rock mixture based on reconstruction of digital image meso–model. J. Railway. Sci. Eng..

